# circ_0014736 induces GPR4 to regulate the biological behaviors of human placental trophoblast cells through miR-942-5p in preeclampsia

**DOI:** 10.1515/med-2023-0645

**Published:** 2023-03-01

**Authors:** Jinlian Ren, Jing Cai

**Affiliations:** Department of Obstetrics, Zhuji Affiliated Hospital of Wenzhou Medical University, Shaoxing, Zhejiang, China; Department of Pathology, Shanghai Jiading District Anting Hospital, No. 1060 Hejing Road, Anting Town, Jiading District, Shanghai, China

**Keywords:** preeclampsia, circ_0014736, miR-942-5p, GPR4

## Abstract

Previous studies have indicated that the development of preeclampsia (PE) involves the regulation of circular RNA (circRNA). However, the role of hsa_circ_0014736 (circ_0014736) in PE remains unknown. Thus, the study proposes to reveal the function of circ_0014736 in the pathogenesis of PE and the underlying mechanism. The results showed that circ_0014736 and GPR4 expression were significantly upregulated, while miR-942-5p expression was downregulated in PE placenta tissues when compared with normal placenta tissues. circ_0014736 knockdown promoted the proliferation, migration, and invasion of placenta trophoblast cells (HTR-8/SVneo) and inhibited apoptosis; however, circ_0014736 overexpression had the opposite effects. circ_0014736 functioned as a sponge for miR-942-5p and regulated HTR-8/SVneo cell processes by interacting with miR-942-5p. Additionally, GPR4, a target gene of miR-942-5p, was involved in miR-942-5p-mediated actions in HTR-8/SVneo cells. Moreover, circ_0014736 stimulated GPR4 production through miR-942-5p. Collectively, circ_0014736 inhibited HTR-8/SVneo cell proliferation, migration, and invasion and induced cell apoptosis through the miR-942-5p/GPR4 axis, providing a possible target for the treatment of PE.

## 1 Introduction

Characterized by proteinuria and hypertension, preeclampsia (PE) affects 3–5% of pregnancies and leads to a wide range of pathophysiological processes, such as inadequate trophoblast invasion, inappropriate maternal vascular destruction, and impaired implantation [1,2]. PE is the major cause of high fetal morbidity and pregnancy-caused death [3]. The increased apoptosis and shallow trophoblastic invasion of cytotrophoblasts are the major triggers of PE [4]. The decreased invasiveness of trophoblasts leads to unsuccessful decidual spiral arteries remodeling and deprived maternal oxygen supply, which leads to the release of antiangiogenic factors, finally causing endothelial cell dysfunction and systemic inflammation [5,6]. Although there are a wide variety of studies on PE, the potential mechanism related to the pathogenesis of PE remains to be explored.

Circular RNA (circRNA) is generated by the back-splicing of precursor mRNA and is highlighted by a covalent bond linking a 5′ cap and 3′ polyadenylation tail, accounting for about 90% of all human transcriptomes [7]. CircRNA is more resistant to exonuclease-induced degradation [8]. Considerable evidence has suggested that circRNA is involved in the pathophysiological process of human diseases, including epithelial–mesenchymal transition (EMT) progress, tumorigenesis, cell apoptosis, and immune responses [9]. At the molecular level, circRNA mediates mRNA expression by sequestering proteins, interfering with pre-mRNA processing or trapping miRNA [10]. Some reports have indicated that the abnormal expression of circRNA in the maternal–fetal interface is related to the mechanism regarding the proliferation, metastasis, and apoptosis of trophoblast cells [11]. Besides, circRNAs are responsible for pregnancy-linked complications [12,13]. circ_0014736, a novel circRNA, is one of the top two upregulated circRNAs in PE placenta tissues, and its expression is associated with some pathways like apoptosis and wnt-signaling [14], suggesting the potential role of circ_0014736 in PE progression. However, no experimental data support the involvement of circ_0014736 in PE.

MicroRNA (miRNA) is a small single-stranded posttranscriptional regulatory molecule that regulates gene expression by promoting their degradation or inhibiting their translation [15]. It has been accepted that the biological processes of various cells, such as cell proliferation, angiogenesis, and cell development, involve the regulation of miRNA. A specific expression pattern of miRNAs has been found in placental tissues and human umbilical vein endothelial cells (HUVECs) [16,17]. Additionally, biological information analysis revealed that miRNA mediated transcriptional regulation, cell cycle, cell adhesion, mitogen-activated protein kinase signaling, and so on, in PE [18,19].

As predicted by online databases, we found that circ_0014736 might bind to miR-942-5p, and G protein-coupled receptor 4 (GPR4) could interact with miR-942-5p. Also, cross-sectional evidence has revealed that miR-942-5p contributes to cell proliferation and motility but impedes cell apoptosis in placenta trophoblast cells [20]. Meanwhile, a recent paper documented that GPR4 was overexpressed in PE placentas and weakened the proliferative and migratory abilities of placenta trophoblast cells [21]. Based on the above data, it is hypothesized that circ_0014736 mediates PE progression by the miR-942-5p/GPR4 pathway; however, the mechanism has not been reported. Thus, the study was organized to assess circ_0014736 expression in PE placenta tissues, explore its role during PE and identify the circRNA/miRNA/mRNA regulatory network in PE development.

## 2 Materials and methods

### 2.1 Clinical specimens

Twenty-eight pregnant women with PE and 28 normal pregnant women (volunteers) were selected from Anting Hospital, Jiading District as research subjects. PE diagnosis was performed according to the stipulation in Williams Obstetrics. Instantly after surgery, tissues were stored at −80°C for further gene expression analysis. The clinical characteristics of the study subjects are shown in Table 1.

**Table 1:** Clinical characteristics of the study samples

Characteristics	Control (*n* = 28)	PE (*n* = 28)	*P*-value
Maternal age (years)	29.78 ± 5.60	30.05 ± 5.31	0.854
Gestational age (week)	38.20 ± 2.66	37.45 ± 3.15	0.340
BMI	26.65 ± 5.61	25.86 ± 4.67	0.569
Systolic blood pressure (mmHg)	118.03 ± 8.36	150.25 ± 18.42	0.000*
Diastolic blood pressure (mmHg)	73.64 ± 8.85	91.72 ± 12.64	0.000*
Neonatal weight (kg)	3.22 ± 0.35	2.62 ± 0.95	0.003*

Values were presented as mean ± SD (Student’s *t*-test). *, *P* < 0.05.

PE, preeclampsia.


**Ethics approval and consent to participate:** The present study was approved by the ethical review committee of Anting Hospital, Jiading District. Written informed consent was obtained from all enrolled patients.
**Consent for publication:** Patients agree to participate in this work

### 2.2 Cell culture

Human placenta trophoblast cells (HTR-8/SVneo) were purchased from Procell (Wuhan, China) and maintained in Roswell Park Memorial Institute-1640 (RPMI-1640; Biosun, Shanghai, China) added with 10% fetal bovine serum (FBS; Biosun) and 1% penicillin/streptomycin (Phygene, Fuzhou, China) at 37°C in a humid incubator with 5% CO_2_.

### 2.3 Cell transfection

According to the user’s manual, plasmids and oligonucleotides were transfected into HTR-8/SVneo cells at 50–80% confluence using FuGENE6 (Roche, Basel, Switzerland). Ribobio Co., Ltd (Guangzhou, China) synthesized the small interfering RNAs against circ_0014736 (si-hsa_circ_0014736#1 5′-TTCTGATGAGGCTGTGCAGAA-3′ and si-hsa_circ_0014736#2 5′-TTTCTGATGAGGCTGTGCAGA-3′), GPR4 (si-GPR4 5′-CGCCATCCCTCTACATCTTTGTCAT-3′), miR-942-5p mimics (miR-942-5p 5′-UCUUCUCUGUUUUGGCCAUGUG-3′), miR-942-5p inhibitors (anti-miR-942-5p 5′-CACAUGGCCAAAACAGAGAAGA-3′), and the respective controls (si-NC, miR-NC, and anti-miR-NC). The overexpression plasmid of circ_0014736 was built by Geneseed Co., Ltd (Guangzhou, China), using the pcD5-ciR vector (pcD-ciR).

**Table 2:** Primers sequences used for qRT-PCR

Name		Sequences (5′–3′)
hsa_circ_0014736	Forward	GAAAAGACAAAGTCCTCGAG
	Reverse	GGATACCTTCTGCACAGC
MEF2D	Forward	TATCAACAACAGCCGAGGCG
	Reverse	TCCGGGCGGGGAGAATAATA
miR-942-5p	Forward	GCGCGCTCTTCTCTGTTTTGGC
	Reverse	GTGCAGGGTCCGAGGT
GPR4	Forward	GCCGTTGTCAAGACCGGG
	Reverse	GGAAATTTCAATGGGGGCAGG
GAPDH	Forward	CAAATTCCATGGCACCGTCA
	Reverse	GACTCCACGACGTACTCAGC
U6	Forward	CTTCGGCAGCACATATACT
	Reverse	AAAATATGGAACGCTTCACG

### 2.4 Quantitative real-time polymerase chain reaction (qRT-PCR)

Total RNA was isolated using Axygen® RNA Miniprep Kit (Corning, Madison, New York) as instructed. After RNA quantity was measured using a UV-3100PC spectrophotometer, high-capacity cDNA synthesis kits (TaKaRa, Dalian, China) were used to reversely transcript RNA. qRT-PCR Mix (TaKaRa) was premixed with cDNA and primers and was then heated on a thermocycler (Stratagene, Santa Clara, CA, USA) to determine the cycle threshold of each sample. The 2^−∆∆Ct^ method was used to analyze gene expression with U6 and glyceraldehyde 3-phosphate dehydrogenase (GAPDH) as internal references. The sequence amplified using the qRT-PCR primer (Table 2) of circ_0014736 contains the head-to-tail splicing sites of circ_0014736, which can distinguish mature circ_0014736 from parental mRNA transcript MEF2D. The forward primer sequence of circ_0014736 is the 5514–5533 bases of the mature sequence, and the reverse primer sequence is the reverse complementary sequence of the 4–21 bases of the mature circ_0014736 sequence.

### 2.5 Identification of circRNA stability

The isolated RNA went through 20 min incubation with RNase R (Tiangen, Beijing, China) at 37°C. Besides, HTR-8/SVneo cells maintained in 12-well plates were co-incubated with Actinomycin D (2 μg/mL; Rechemscience, Shang, China) for 0, 6, 12, and 24 h. Finally, qRT-PCR was applied to detect circ_0014736 expression. Linear myocyte enhancer factor 2D (MEF2D) served as a control gene reference.

### 2.6 3-(4,5-Dimethylthazol-2-yl)-2,5-diphenyltetrazolium bromide (MTT) assay

HTR-8/SVneo cells were passaged in 96-well microplates (5,000 cells per well) and cultured for 16 h. Then, si-hsa_circ_0014736#1, si-hsa_circ_0014736#2, si-NC, hsa_circ_0014736, pcD-ciR, miR-942-5p, miR-NC, anti-miR-942-5p, anti-miR-NC, and si-GPR4 were transfected into the cells and cultured for 0, 1, 2, 3, and 4 days. The cells were incubated with MTT solution (Seebio Biotech, Shanghai, China) and dimethyl sulfoxide (Seebio Biotech) successively. At last, samples were analyzed by a microplate reader (BioTek, Winooski, VT, USA) with an absorbance of 570 nm.

### 2.7 5-Ethynyl-2′-deoxyuridine (EdU) assay

HTR-8/SVneo cells transfected with test compounds went through 48 h culture in 12-well plates. The cells were digested and seeded in 96-well plates supplemented with EdU-labeled medium. Then, cell proliferation was confirmed by analyzing the number of EdU-positive cells using an EdU staining kit (Ribobio) following the guidebook. In addition, the immunostainings were captured under a fluorescence microscope (Olympus, Tokyo, Japan).

### 2.8 Flow cytometry analysis

After various transfections, HTR-8/SVneo cells were collected and washed using phosphate buffer solution (PBS; Phygene). Annexin V-FITC apoptosis detection kit (Solarbio, Beijing, China) was then performed to detect cell apoptosis in accordance with the guidebook. In short, the cells were suspended in binding buffer, and cell concentration was adjusted to 1 × 10^5^ cells per 100 μL PBS. The cells were incubated with Annexin V-FITC and propidium iodide in the dark. At last, a flow cytometer (Thermo Fisher, Waltham, MA, USA) was applied to analyze the samples.

### 2.9 Wound-healing assay

Cells were placed in six-well plates and treated with test compounds. When the low surface of the culture plates was filled with the cells, cell wounds were created by using pipette tips (Baisai, Shanghai, China). After removing floated cells and debris, the cells were maintained in serum-free medium for 24 h. The width of the scratch gaps was measured at 0 and 24 h after serum-free cell culture under a low-power microscope (40× magnification; Olympus).

### 2.10 Transwell assay

The assay was carried out on HTR-8/SVneo cells grown in 12-well chambers added with 100 μL Matrigel (Corning). In brief, 5 × 10^4^ cells were mixed with serum-free RPMI-1640 (Biosun) and then added into the upper insert of each chamber, while the medium containing 15% FBS (Biosun) was placed into the lower inserts to serve as chemoattractants. After 24 h, the cells that went through the membranes were fixed and stained with methanol (Seebio Biotech) and crystal violet (Seebio Biotech), respectively. The cells in six representative fields were determined under a high-power microscope (100× magnification; Olympus).

### 2.11 Western blot analysis

Total proteins from HTR-8/SVneo cells were extracted by using western cell lysis buffer (Sangon, Shanghai, China). The lysates were mixed with loading buffer (Thermo Fisher) and heated at 95°C to denature the proteins. Then, 20 μg of protein was placed in the holes of SurePAGE gels (Sangon) and electrophoresed through XCell4 SureLock Electrophoresis System (Thermo Fisher). After the proteins were wet-transferred onto nitrocellulose membranes, skimmed milk was used to block aspecific signals. The primary antibodies specific for proliferating cell nuclear antigen (PCNA), cleaved-caspase 3 (c-caspase 3), matrix metalloprotein 9 (MMP-9), GPR4 and GAPDH were used to incubate the membranes at a dilution of 1:1,000. Subsequent steps were carried out using a Western kit (Sangon). GAPDH was used to normalize protein expression. All antibodies were provided by Cusabio Biotech (Wuhan, China) or Sigma (St Louis, MO, USA).

### 2.12 Dual-luciferase reporter assay

Circular RNA interactome (https://circinteractome.nia.nih.gov/index.html) and targetscan online databases (http://www.targetscan.org/vert_71/) were conducted to predict the complementary sites of miR-942-5p with circ_0014736 and GPR4. Then, the sequences of circ_0014736 and GPR4 3′-untranslated region (3′-UTR) carrying the binding sites of miR-942-5p were amplified by qRT-PCR to build the wild-type (WT) plasmids (hsa_circ_0014736-WT and GPR4 3′-UTR-WT). The complementary sites within circ_0014736 and GPR4 3′-UTR were mutated by GenScript Biotech Corporation (Nanjing, China) to build the mutant (MUT) plasmids including hsa_circ_0014736-MUT and GPR4 3′-UTR-MUT. For cell transfection, the reporter vectors were mixed with FuGENE6 (Roche) and added into 24-well plates together with miR-942-5p or miR-NC. After 48 h of transfection, the luciferase activity of HTR-8/SVneo cells was monitored using Dual-Lucy Assay Kit (Solarbio).

### 2.13 RNA immunoprecipitation (RIP) assay

HTR-8/SVneo cells were collected and lysed using RIP lysis buffer (Sangon) containing RNasin (TaKaRa). The proteins that bound to the antibodies against AGO2 (anti-AGO2) and IgG (anti-IgG) were enriched by using a Magna RIP kit (Millipore, Billerica, MA, USA) following the standard procedures. qRT-PCR was applied to quantify circ_0014736 and miR-942-5p expression.

### 2.14 Biotin-labeled pull-down assay

GenePharma Company (Shanghai, China) provided biotin-labeled miR-942-5p (Biotin-miR-942-5p) and miR-NC (Biotin-NC). This assay was carried out on HTR-8/SVneo cells. Briefly, the cells were treated with the above probes and cultured for 48 h. Then, the cells were lysed and incubated with Streptavidin MagneSphere (Sigma) for 4 h. At last, circ_0014736 and GPR4 expression were monitored by qRT-PCR.

### 2.15 Statistical analysis

All data were obtained from three independent biological experiments, and data analysis was conducted using GraphPad Prism software. Results are expressed as mean ± standard deviation. Difference analysis was performed by Mann–Whitney *U*-test or Student’s *t*-test between the two groups and implemented by one-way analysis of variance among three or more groups. *P* < 0.05 indicated a statistical difference.

## 3 Results

### 3.1 circ_0014736 expression was upregulated in PE placenta tissues

**Figure 1:**
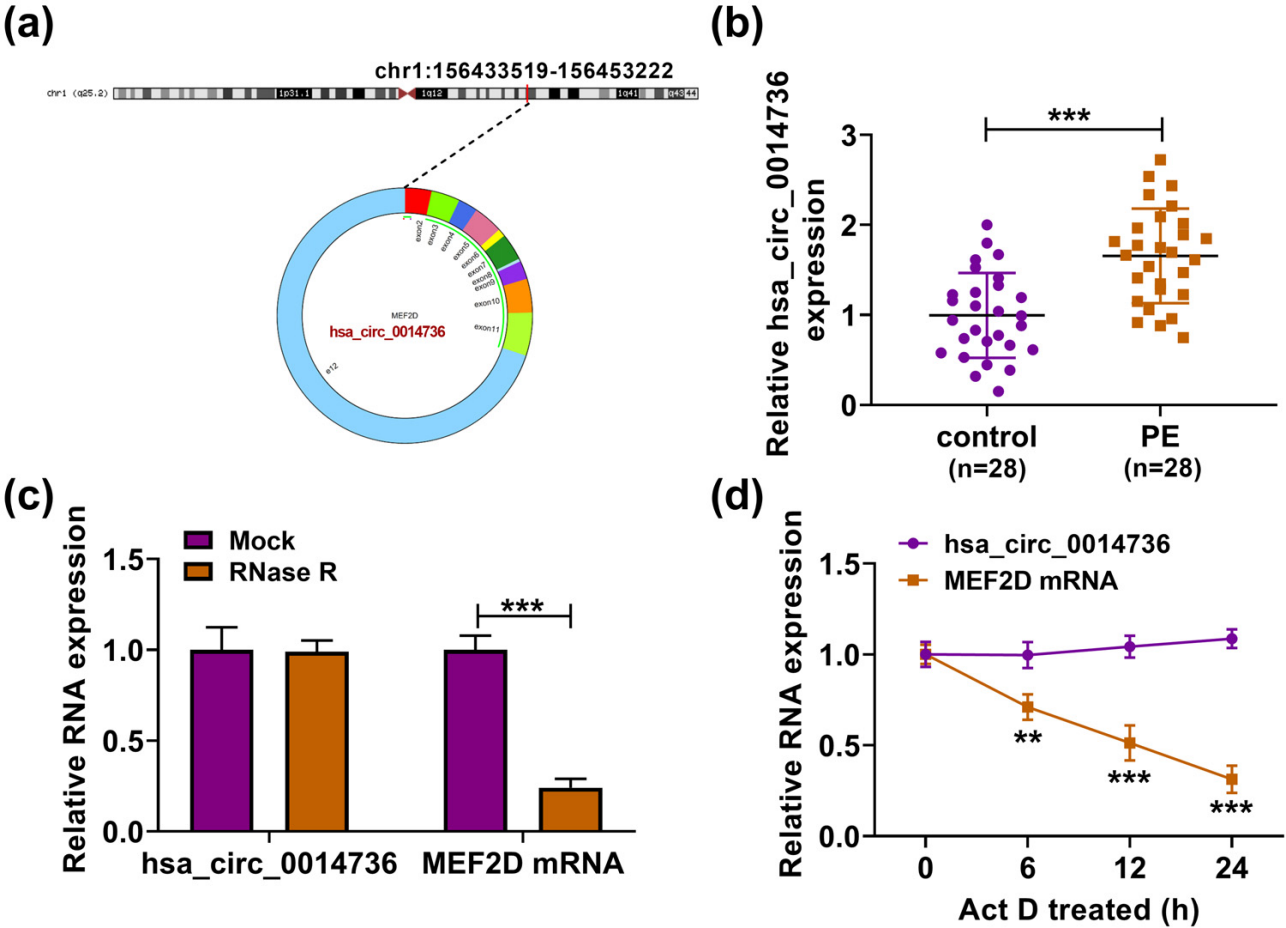
Expression of circ_0014736 in PE tissues. (a) circ_0014736 was formed by the cyclization of exons 2–12 of the MEF2D gene. (b) circ_0014736 expression was detected by qRT-PCR in PE placenta tissues (*N* = 28) and normal placenta tissues (*N* = 28). (c and d) Stability of circ_0014736 was identified by RNase R and Actinomycin D treatment assays. **P < 0.01 and ***P < 0.001. Three independent duplicate tests were performed in (c) and (d).

circ_0014736 is located in chr1: 156433519–156453222 and is formed by the cyclization of exons 2–12 of the MEF2D gene (Figure 1a). Currently, there are no data regarding the role of circ_0014736 in the pathological genesis of PE. To analyze the role of circ_0014736 in PE, qRT-PCR was performed to detect circ_0014736 expression in PE placenta tissues. As shown in Figure 1b, circ_0014736 was overexpressed in PE placenta tissues when compared with normal placenta tissues. Subsequently, RNase R treatment did not affect the expression of circ_0014736 but significantly reduced MEF2D expression (Figure 1c). Also, the data from Figure 1d showed that the transcript half-life of circ_0014736 exceeded 24 h, although that of linear MEF2D was about 12 h. Taken together, these data indicated the pathological genesis of PE might involve circ_0014736.

### 3.2 circ_0014736 inhibited the proliferation, migration, and invasion and induced apoptosis of HTR-8/SVneo cells

**Figure 2:**
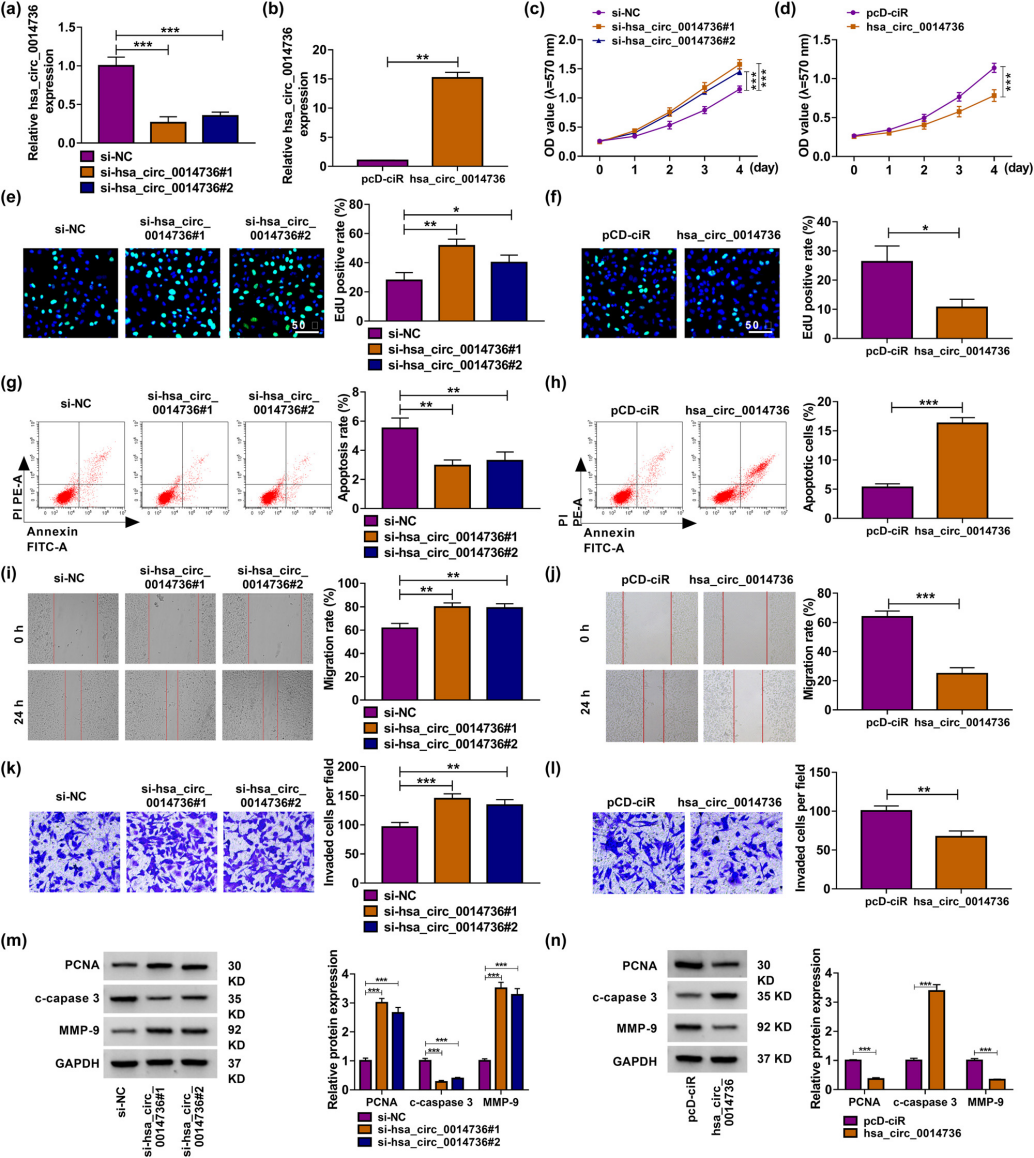
Effects of circ_0014736 on the proliferation, migration, invasion, and apoptosis of HTR-8/SVneo cells. (a and b) Efficiency of circ_0014736 knockdown and overexpression was determined by qRT-PCR in HTR-8/SVneo cells. HTR-8/SVneo cells were transfected with si-NC, si-hsa_circ_0014736#1, and si-hsa_circ_0014736#2 (c, e, g, i, k, and m), or pcD-ciR and hsa_circ_0014736 (d, f, h, j, l, and n). (c–f) Cell proliferation was investigated by MTT and EdU assays. (g and h) Cell apoptosis was quantified by flow cytometry analysis. (i–l) Migration and invasion were evaluated by wound-healing and transwell assays, respectively. (m and n) Expression of PCNA, c-caspase 3, and MMP-9 was checked by western blot. *P < 0.05, **P < 0.01, and ***P < 0.001. Three independent duplicate tests were performed in each experiment.

The study then explored the function of circ_0014736 in the biological behaviors of HTR-8/SVneo cells, including cell proliferation, migration, invasion, and apoptosis, by silencing and overexpressing circ_0014736. The data from Figure 2a and b showed the high efficiency of circ_0014736 knockdown and overexpression. Subsequently, circ_0014736 depletion increased HTR-8/SVneo cell proliferation, but circ_0014736 overexpression displayed the opposite effects (Figure 2c–f). circ_0014736 absence inhibited the apoptosis of HTR-8/SVneo cells, whereas circ_0014736 introduction promoted cell apoptosis (Figure 2g and h). Consistently, the migration and invasion of HTR-8/SVneo cells were enhanced after circ_0014736 depletion but weakened by the enforced expression of circ_0014736 (Figure 2i–l). The study further detected the expression of proliferation-related PCNA, apoptosis-related c-caspase 3, and metastasis-related MMP-9 in the HTR-8/SVneo cells. As expected, circ_0014736 knockdown increased the expression of PCNA and MMP-9 and decreased c-caspase 3 expression; however, ectopic circ_0014736 expression had the opposite effects (Figure 2m and n). Thus, the above data manifested that circ_0014736 could induce HTR-8/SVneo cell dysfunction.

### 3.3 circ_0014736 acted as a sponge for miR-942-5p

**Figure 3:**
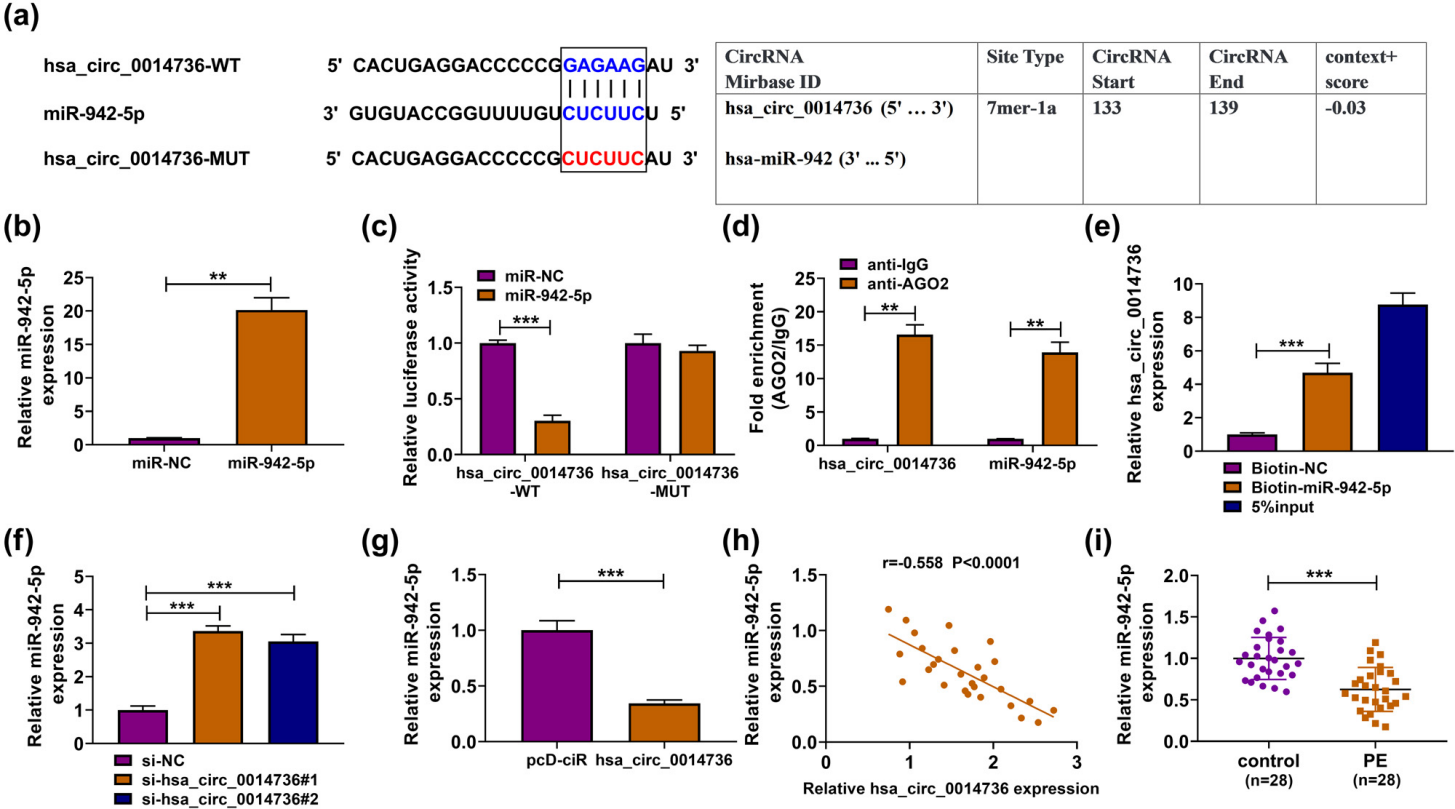
circ_0014736 interacted with miR-942-5p. (a) Binding sites of circ_0014736 for miR-942-5p. (b) Efficiency of miR-942-5p overexpression was determined by qRT-PCR in HTR-8/SVneo cells. (c–e) Dual-luciferase reporter, RIP, and RNA pull-down assays were used to identify the interaction of circ_0014736 and miR-942-5p. (f and g) Effects of circ_0014736 knockdown and overexpression on miR-942-5p expression were analyzed by qRT-PCR in HTR-8/SVneo cells. (h) Correlation between circ_0014736 and miR-942-5p expression was analyzed by Spearman correlation analysis in PE placenta tissues. (i) MiR-942-5p expression was determined by qRT-PCR in PE placenta tissues (*N* = 28) and normal placenta tissues (*N* = 28). **P < 0.01 and ***P < 0.001. Three independent duplicate tests were performed in (c–g).

MiRNA with the potential to bind to circ_0014736 was analyzed in this part. As a result, it was found that circ_0014736 potentially targeted miR-942-5p (Figure 3a). To analyze the potential binding relationship of circ_0014736 and miR-942-5p, the efficiency of miR-942-5p overexpression was detected. As shown in Figure 3b, the transfection with miR-942-5p mimics significantly increased miR-942-5p expression in HTR-8/SVneo cells, which indicated the high efficiency of miR-942-5p mimics in increasing miR-942-5p expression. Then, we found that miR-942-5p overexpression inhibited the luciferase activity of the WT reporter plasmid of hsa_circ_0014736 but not the luciferase activity of mutant reporter plasmid of hsa_circ_0014736 (Figure 3c). The RIP assay showed that both circ_0014736 and miR-942-5p were significantly enriched in the anti-AGO2 group when compared with the anti-IgG group in HTR-8/SVneo cells (Figure 3d). Moreover, biotin-labeled miR-942-5p could dramatically enrich circ_0014736 when compared with circ_0014736 expression in the control groups (Figure 3e). The above findings confirmed that circ_0014736 bound to miR-942-5p in HTR-8/SVneo cells. Subsequent data showed that circ_0014736 negatively regulated miR-942-5p expression. For instance, circ_0014736 depletion increased miR-942-5p expression, but circ_0014736 overexpression decreased the expression of miR-942-5p (Figure 3f and g). In support, Spearman correlation analysis showed the negative correlation of circ_0014736 and miR-942-5p expression in PE placenta tissues (Figure 3h). Comparatively, miR-942-5p was weakly expressed in PE placenta tissues (Figure 3i). Based on these data, miR-942-5p was employed as the follow-up study subject.

### 3.4 miR-942-5p attenuated the effects of circ_0014736 overexpression on HTR-8/SVneo cell processes

**Figure 4:**
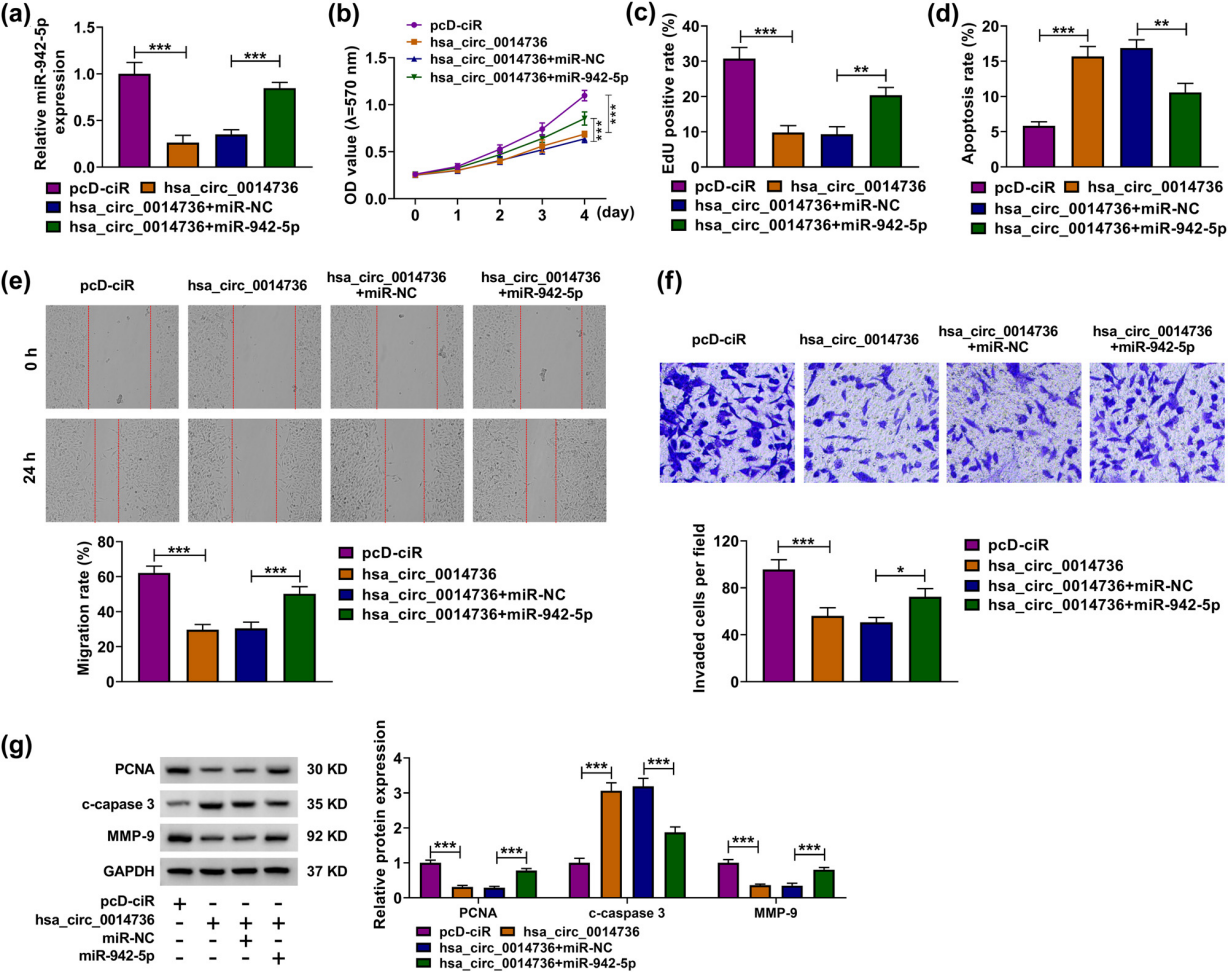
Effects of circ_0014736 and miR-942-5p overexpression on the proliferation, migration, invasion, and apoptosis of HTR-8/SVneo cells. (a–g) HTR-8/SVneo cells were transfected with pcD-ciR, hsa_circ_0014736, hsa_circ_0014736 + miR-NC, or hsa_circ_0014736 + miR-942-5p, and miR-942-5p expression was detected by qRT-PCR (a), cell proliferation by MTT and EdU assays (b and c), cell apoptosis by flow cytometry analysis (d), cell migration by wound-healing assay (e), cell invasion by transwell assay (f), and the protein expression of PCNA, c-caspase 3, and MMP-9 by western blot analysis (g). *P < 0.05, **P < 0.01, and ***P < 0.001. Three independent duplicate tests were performed in each experiment.

Whether miR-942-5p participated in circ_0014736-induced HTR-8/SVneo cell disorder was analyzed in this part. Figure 4a first showed that circ_0014736 overexpression reduced miR-942-5p expression, whereas the effect was relieved after transfection with miR-942-5p. Then, the inhibitory impact of the enforced circ_0014736 expression on HTR-8/SVneo cell proliferation was remitted after miR-942-5p introduction (Figure 4b and c). The addition of circ_0014736 expression promoted HTR-8/SVneo cell apoptosis, which was rescued by the increased expression of miR-942-5p (Figure 4d). A similar pattern of data among various groups was also found from wound-healing and transwell assays (Figure 4e and f). Consistently, miR-942-5p introduction restored the decreased expression of PCNA and MMP-9 and increased c-caspase 3 expression caused by circ_0014736 (Figure 4g). Thus, these results demonstrated that circ_0014736-induced dysfunction of HTR-8/SVneo cells involved miR-942-5p.

### 3.5 circ_0014736 modulated GPR4 expression through miR-942-5p

**Figure 5:**
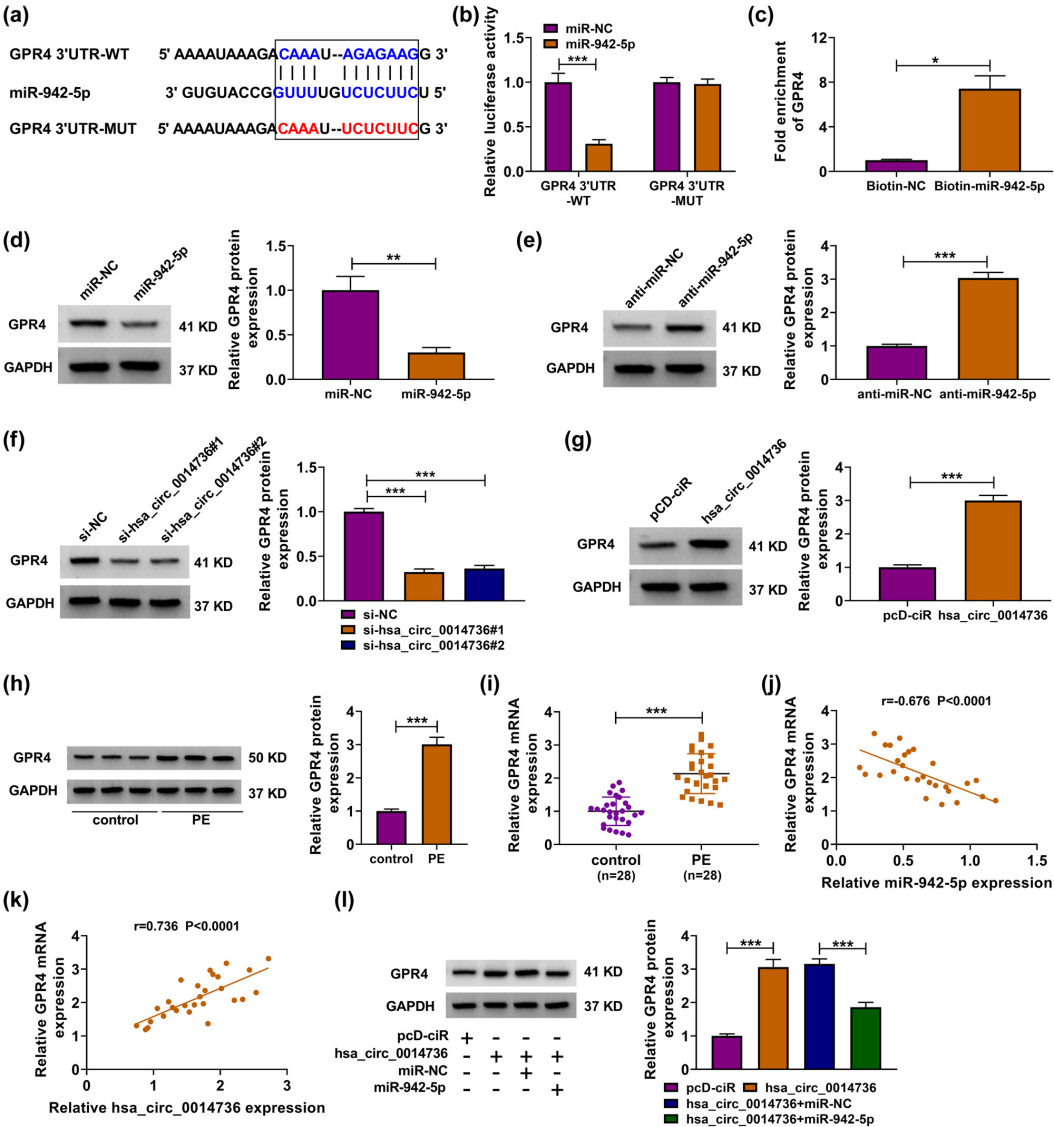
circ_0014736 regulated GPR4 expression by miR-942-5p. (a) Binding sites of miR-942-5p for GPR4 3′-UTR. (b and c) Dual-luciferase reporter and RNA pull-down assays were performed to determine the interaction of GPR4 and miR-942-5p. (d and e) Effects of miR-942-5p overexpression and knockdown on GPR4 protein expression were investigated by western blot. (f and g) Western blot analysis was used to analyze the effects of circ_0014736 silencing and overexpression on GPR4 protein expression. (h and i) Protein and mRNA expression of GPR4 were detected by qRT-PCR and western blot analysis, respectively, in PE placenta tissues and normal placenta tissues. (j and k) Spearman correlation analysis was employed to determine the correlation between GPR4 and miR-942-5p or circ_0014736 in PE tissues. (l) Impacts between circ_0014736 and miR-942-5p overexpression on GPR4 protein expression were analyzed by western blot analysis. *P < 0.05, **P < 0.01, and ***P < 0.001. Three independent duplicate tests were performed in (b–h and l).

To determine whether GPR4 participated in the regulation of miR-942-5p in HTR-8/SVneo cell processes, we predicted the binding sites of miR-942-5p for GPR4 using targetscan online database. As shown in Figure 5a, GPR4 carried the complementary sites of miR-942-5p. Dual-luciferase reporter assay displayed that the luciferase activity of the WT reporter plasmid of GPR4 3′-UTR was inhibited after transfection with miR-942-5p mimics; however, the luciferase activity of mutant GPR4 3′-UTR had no response to miR-942-5p overexpression (Figure 5b). RNA pull-down assay showed that biotinylated miR-942-5p significantly enriched GPR4 in HTR-8/SVneo cells when compared with the controls (Figure 5c). The above results suggested that GPR4 was a target mRNA of miR-942-5p. Thus, GPR4 was employed in the following assays. Subsequently, we found that miR-942-5p introduction reduced GPR4 expression (Figure 5d). GPR4 expression was upregulated after miR-942-5p depletion (Figure 5e). Consistently, the study corroborated that circ_0014736 positively regulated GPR4 expression (Figure 5f and g). For instance, circ_0014736 knockdown decreased GPR4 expression, but circ_0014736 overexpression had the opposite effect. The data from Figure 5h and i showed that GPR4 was augmented in PE tissues in comparison with the controls. In support, GPR4 was negatively correlated with miR-942-5p expression but positively with circ_0014736 in PE tissues (Figure 5j and k). As expected, the addition of circ_0014736 expression elevated GPR4 expression, which was attenuated after miR-942-5p overexpression (Figure 5l). Taken together, these data demonstrated that circ_0014736 could mediate GPR4 expression through miR-942-5p.

### 3.6 GPR4 knockdown relieved the effects of miR-942-5p depletion on the proliferation, migration, invasion, and apoptosis of HTR-8/SVneo cells

**Figure 6:**
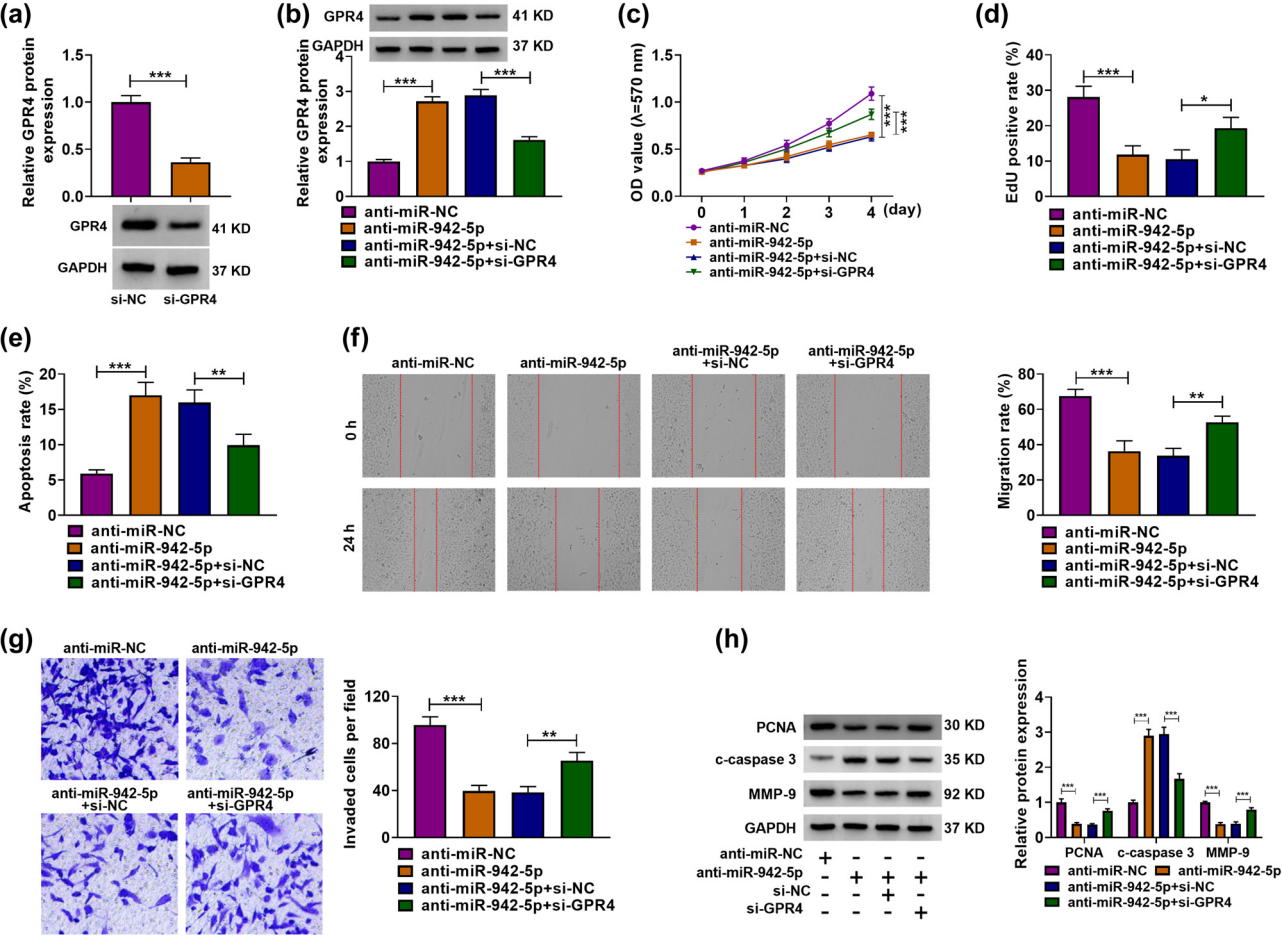
Effects between miR-942-5p inhibitors and GPR4 knockdown on the proliferation, migration, invasion, and apoptosis of HTR-8/SVneo cells. (a) Knockdown efficiency of GPR4 was determined by western blot in HTR-8/SVneo cells. (b–h) HTR-8/SVneo cells were transfected with anti-miR-NC, anti-miR-942-5p, anti-miR-942-5p + si-NC, or anti-miR-942-5p + si-GPR4, and protein expression of GPR4, PCNA, c-caspase 3, and MMP-9 was determined by western blot (b and h), cell proliferation by MTT and EdU assays (c and d), apoptosis by flow cytometry analysis (e), cell migration by wound-healing assay (f), and cell invasion by transwell assay (g). *P < 0.05, **P < 0.01, and ***P < 0.001. Three independent duplicate tests were performed in each experiment.

The study continued to analyze whether miR-942-5p regulated HTR-8/SVneo cell processes by interacting with GPR4. Before that, we identified the efficiency of GPR4 knockdown (Figure 6a). Subsequently, the reduced expression of miR-942-5p promoted GPR4 protein production, which was reversed after GPR4 knockdown (Figure 6b). The proliferation of HTR-8/SVneo cells was inhibited after miR-942-5p silencing, whereas the decreased expression of GPR4 restored the effect (Figure 6c and d). Comparatively, the transfection with miR-942-5p inhibitors promoted cell apoptosis, but the decreased expression of GPR4 remitted the effect (Figure 6e). Besides, the data from Figure 6f and g also displayed that the decrease in GPR4 expression rescued the inhibitory impacts of miR-942-5p inhibitors on the migration and invasion of HTR-8/SVneo cells. In support, the decreased expression of PCNA and MMP-9 and the increased expression of c-caspase 3 by miR-942-5p inhibitors were remitted after GPR4 depletion (Figure 6h). By and large, these data manifested that miR-942-5p-triggered HTR-8/SVneo cell disorder involved GPR4.

## 4 Discussion

In contrast to late PE, early PE is more associated with increased severity of complications [22]. At present, the mechanisms behind PE progression are still poorly understood due to many complex pathophysiological factors in the process. Increasing evidence has confirmed the association of circRNA with PE progression [11]. HTR-8/SVneo cells are commonly used to investigate trophoblast biology and placental function, contributing to understanding the pathophysiological mechanisms of diseases related to inadequate placentation invasiveness in PE [23]. Under the above research background, the study was designed to explore the role of circ_0014736 in PE progression using HTR-8/SVneo cells. As a result, we found that circ_0014736 expression was upregulated in PE placenta tissues. circ_0014736 inhibited HTR-8/SVneo cell proliferation, migration, invasion, and induced apoptosis. In terms of mechanism, circ_0014736 induced GPR4 production through miR-942-5p.

Numerous data suggested the pathophysiological mechanisms of PE involved circRNA. For instance, circ_0001438 expression was enhanced in PE specimens, and circ_0001438 silencing promoted cell proliferation and motility but impeded inflammation through miR-942 in the placenta trophoblast cells [20]. CircRNA adenylate kinase 2 weakened trophoblast cell proliferation and metastasis, and the underneath mechanism was attributed to the miR-454-3p/thrombospondin 2 pathway [24]. Besides, circRNA trinucleotide repeat containing 18 sponged miR-762 to induce grainyhead like transcription factor 2, enhancing trophoblast cell migration and EMT [25]. In this work, circ_0014736, located on chr1: 156433519–156453222 and formed by the cyclization of exons 2–12 of the MEF2D gene, was discovered for the first time to regulate placenta trophoblast cell processes. Herein, circ_0014736 was upregulated in PE placentas when compared to normal placental tissues. Functional studies showed that circ_0014736 knockdown contributed to cell proliferation and metastasis but reduced cell apoptotic rate; however, circ_0014736 overexpression showed the opposite effects. These data indicated that circ_0014736 might act as a promoter in PE progression.

Given that circRNA generally acted as a sponge of miRNA to function in different types of diseases, we continued to analyze circ_0014736-related miRNAs. Through the prediction of online databases (circular RNA interactome and targetscan) and verification of mechanism assays, miR-942-5p was chosen as the target miRNA of circ_0014736. MiR-942-5p, a newly detected miRNA, is responsible for the mechanisms of multiple diseases, such as septic acute kidney injury [26], cancers [27,28], atherosclerosis [29], and attention deficit hyperactivity disorder [30]. Recently, some investigators found that miR-942-5p was poorly expressed in women with PE in comparison with women with uncomplicated pregnancies, and its depletion inhibited extravillous trophoblast cell viability and HUVEC angiogenesis [31]. In this work, miR-942-5p was weakly expressed in PE tissues when compared to normal placentas, and miR-942-5p contributed to cell proliferation and motility and repressed cell apoptosis in HTR-8/SVneo cells, which were in line with the reports from Li and his colleagues [20]. Besides, our data suggested that circ_0014736 accelerated the dysfunction of HTR-8/SVneo cells by binding to miR-942-5p.

GPR4, a proton-sensing receptor, is a member of proton-sensing G-protein coupled receptor family that helps cells sense extracellular acidification [32]. As reported, GPR4 participates in the immune response by recruiting monocytes and neutrophils [33,34]. Besides, GPR4 regulated cell metastatic ability and apoptosis in some types of cancers [35,36]. Recent studies indicated that GPR4-linked G protein-coupled receptors were associated with embryonic gastrulation [37]. Previous analysis also suggested that sphingosylphosphorylcholine-induced endothelial tube formation involved the regulation of GPR4 [38]. Another research suggested that GPR4 controlled central blood pressure through interaction with the renin-angiotensin system [39]. In this respect, Qi et al. have reported that GPR4 is highly expressed in HTR8/SVneo cells under acidosis and hypoxia environments [21]. In the present study, GPR4 was verified as a target mRNA of miR-942-5p and negatively regulated by miR-942-5p. Consistent with the published data [21], GPR4 was overexpressed in PE placenta tissues, and its knockdown promoted cell proliferation and migration in HTR8/SVneo cells. Beyond that, our data also found that GPR4 contributed to cell apoptosis and inhibited cell invasion in HTR8/SVneo cells.

Based on the above results, we further analyzed the association between circ_0014736 and GPR4. As a result, the study found that circ_0014736 knockdown reduced GPR4 expression, whereas high circ_0014736 expression had the opposite effect. Also, we found a negative correlation of circ_0014736 expression with GPR4 expression. Rescue assay further showed that circ_0014736-induced upregulation of FPR4 was relieved by the increased expression of miR-942-5p. Thus, circ_0014736 induced HTR8/SVneo cell dysfunction through the miR-942-5p/GPR4 pathway.

Some limitations should be considered when evaluating our findings. First, HTR8/SVneo cells used in this study contained a heterogeneous population of trophoblast and stromal cells [40], and other cell lines, such as BeWo, JEG-3, and JAR, should be chosen to evaluate the new mechanism. Additionally, the findings were only testified *in vitro*, and the mouse model of PE needed to be established to further validate the new mechanism.

## 5 Conclusion

**Figure 7:**
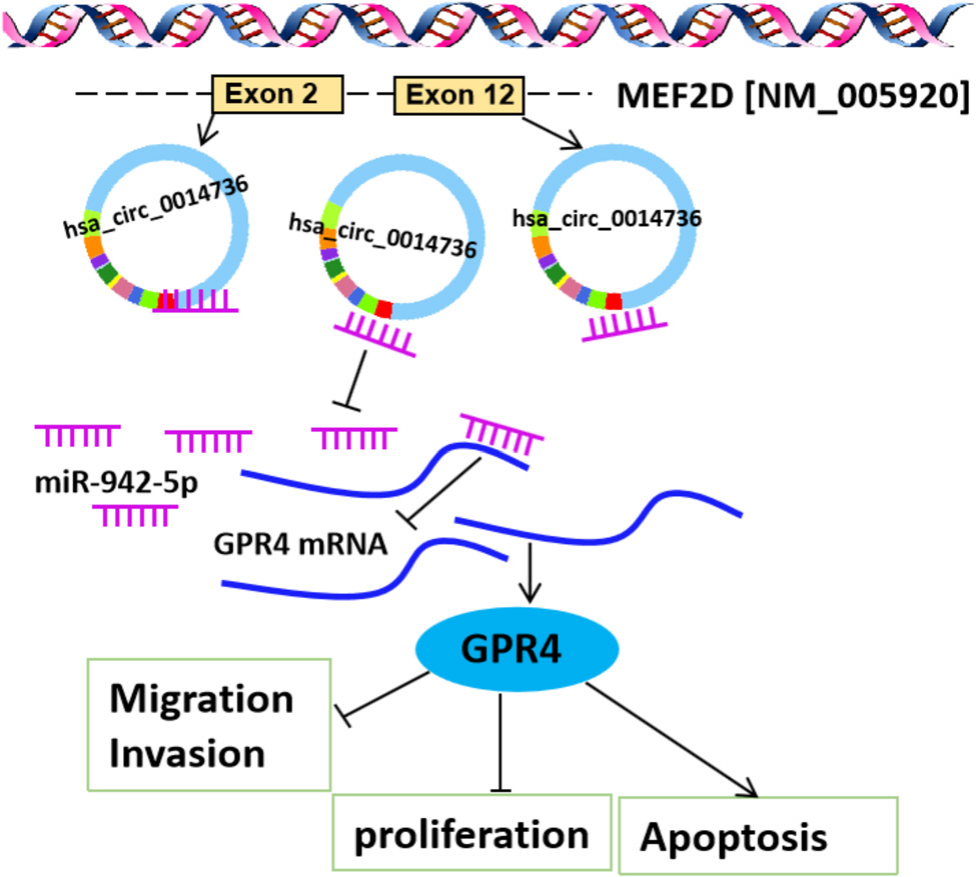
Schematic diagram showed the mechanism of circ_0014736 regulating human placental trophoblast cell dysfunction in PE.

Taken together, the dysfunction of HTR8/SVneo cells in PE involved the high expression of circ_0014736. The ectopic expression of circ_0014736 induced GPR4 production in a miR-942-5p-dependent manner, thereby inhibiting the proliferation, migration, and invasion of HTR8/SVneo cells and inducing cell apoptosis (Figure 7). The novel pathway highlights a new regulatory mechanism for circ_0014736 in PE pathogenesis. Additionally, the study also suggests the potential of circ_0014736 as a therapeutic target for PE. In particular, the inhibitors of circ_0014736 or GPR4 and the mimics of miR-942-5p are potential therapeutic agents for PE. However, due to lack of funding, more evidence showing the application of circ_0014736, miR-942-5p, or GPR4 in the treatment of PE needs to be explained in future studies.

## Abbreviations

Biotin-miR-942-5p: biotin-labeled miR-942-5p
circRNA: circular RNA
EdU: 5-Ethynyl-2′-deoxyuridine
GPR4: G protein-coupled receptor 4
PE: preeclampsia
RIP: RNA immunoprecipitation
WT: wild-type
3′-UTR: 3′-untranslated region
